# *Blastocystis* sp. Infection Mimicking Clostridium Difficile Colitis

**DOI:** 10.1155/2016/7264387

**Published:** 2016-05-09

**Authors:** Gaby S. Gil, Shobhana Chaudhari, Ahmed Shady, Ana Caballes, Joe Hong

**Affiliations:** ^1^Department of Internal Medicine, Metropolitan Hospital Center, New York Medical College, 1901 First Avenue, New York, NY 10029, USA; ^2^Department of Psychiatry, Metropolitan Hospital Center, New York Medical College, 1901 First Avenue, New York, NY 10029, USA

## Abstract

We report an unusual case of severe diarrhea related to* Blastocystis* sp. infection in a patient with end stage renal disease on hemodialysis. The patient was admitted due to profuse diarrhea associated with fever and leukocytosis. Pertinent stool work-up such as leukocytes in stool, stool culture, clostridium difficile toxin B PCR, and serology for hepatitis A, hepatitis B, and hepatitis C and cytomegalovirus screening were all negative. Ova and parasite stool examination revealed* Blastocystis* sp. The patient was given intravenous metronidazole with clinical improvement by day three and total resolution of symptoms by day ten.

## 1. Introduction

In comparison to immunocompetent individuals,* Blastocystis* sp. infection is an important cause of severe diarrhea in the immunocompromised individuals. The self-limited diarrheic nature of this ubiquitous and benign parasite in the general population may halt the need in trying to identify this pathogen in a timely manner as a possible cause of severe diarrhea in immunosuppressed patients [1, 2].

Little is known about the occurrence of sepsis as a manifestation of* Blastocystis* sp. infection in symptomatic patients with deranged immunity, since this is usually a commensal parasite. Infection with other enteral pathogens needs to be ruled out first in order to start a plan of care tailored to treat this microorganism. Nowadays, distinct subtypes and degree of pathogenicity play an important role in the severity of the clinical course in vulnerable patients infected by* Blastocystis* sp. Metronidazole is the medication of choice since it is safe and effective for treating this infection [3].

## 2. Case Presentation

The patient, a forty-seven-year-old male from Guinea, West Africa, presented to the emergency room with one-day history of fever and profuse diarrhea. His past medical history was significant for end stage renal disease requiring hemodialysis, hypertension, and type 2 diabetes mellitus. He reported an exposure to antibiotics two months prior to his current admission.

His vital signs included blood pressure of 166/106 mmHg, heart rate of 136 bpm, temperature of 100.7 F, and respiratory rate of 16 breaths/min. Physical examination was remarkable for generalized maculopapular rash and abdominal tenderness. The rest of the physical evaluation was unremarkable.

Pertinent laboratory analysis showed a white blood cell count of 27,000/mm^3^ with left shift, hemoglobin of 11.2 g/dL, blood urea nitrogen of 68 mg/dL, and creatinine of 6.6 mg/dL. Blood and urine cultures, hepatitis A and hepatitis B antigens, hepatitis C antibody, and cytomegalovirus antigen in the blood were all unremarkable. Stool work-up including clostridium difficile toxin B PCR, leukocytes in stool, stool culture, and cryptosporidium were negative. Ova and parasite stool examination revealed the presence of multiple microorganisms identified as* Blastocystis* sp.

Our patient was started on intravenous fluid hydration and antibiotic therapy with intravenous metronidazole. His diarrhea frequency lessened and white blood cell count improved three days after the initiation of therapy. The patient was discharged on oral metronidazole in light of the presence of* Blastocystis* sp. with total resolution of symptoms by day ten.

## 3. Discussion

End stage renal disease (ESRD) represents a state of deranged immunity that predisposes to numerous complications. Intestinal parasites are associated with increased morbidity and mortality in this population. The protozoa* Blastocystis* sp. is a universal parasite found in stool analysis in the general population. Its prevalence is 1.5–10% in developed countries and 30–50% in developing countries [1–5]. Transmission is commonly through a fecal-oral route with a variety of clinical manifestations.

*Blastocystis* sp. infection is overall self-limited. Fever is usually absent, and urticarial lesions with eosinophilia could be seen only in a few cases. However, in immunocompromised individuals,* Blastocystis* sp. can cause severe diarrhea associated with elevation of white blood cells and fever possibly secondary to the invasive nature of different virulent subtypes. A high index of suspicion for other enteric pathogens has to be taken into account and proper work-up should be made in order to promptly rule out these organisms [3, 5–10].

Cases of invasive intestinal infection by* Blastocystis* sp. in immunocompetent individuals have suggested a pathogenic potential. Genotype variability and various microorganism subtypes have been reported, and each one carries a distinct degree of pathogenicity. Certain subtypes, such as subtype four and subtype seven, have been linked with severe infection, including extraintestinal manifestations in immunocompetent and immunosuppressed patients [8–10].

To our knowledge, this is the first reported case of severe infection by* Blastocystis* sp. in a patient with end stage renal disease on hemodialysis with a satisfactory response to metronidazole.

Clinicians should be aware of the importance of routine stool examination for parasites in patients with renal derangement and acute diarrhea. Treatment for* Blastocystis* sp. is recommended in symptomatic cases with severe diarrhea, particularly in populations with impaired immunity. Metronidazole appears to be the most effective medication, but trimethoprim-sulfamethoxazole is also effective [3].

Simple hygiene measures such as handwashing with water and soap and avoiding contaminated food are still the best way to prevent infectious diarrhea.

Further stool or blood analysis is needed for symptomatic cases infected by* Blastocystis* sp. in order to identify any of these pathogenic subtypes and tailor a prompt plan of care.
